# Translational Medicine and Patient Safety in Europe: TRANSFoRm—Architecture for the Learning Health System in Europe

**DOI:** 10.1155/2015/961526

**Published:** 2015-10-11

**Authors:** Brendan C. Delaney, Vasa Curcin, Anna Andreasson, Theodoros N. Arvanitis, Hilde Bastiaens, Derek Corrigan, Jean-Francois Ethier, Olga Kostopoulou, Wolfgang Kuchinke, Mark McGilchrist, Paul van Royen, Peter Wagner

**Affiliations:** ^1^King's College London, London SE1 3QD, UK; ^2^Karolinska Institutet, 14183 Stockholm, Sweden; ^3^University of Warwick, Coventry CV4 7AL, UK; ^4^University of Antwerp, 2610 Antwerp, Belgium; ^5^Royal College of Surgeons of Ireland, Dublin 2, Ireland; ^6^INSERM, 6 Paris, France; ^7^University of Düsseldorf, 40225 Düsseldorf, Germany; ^8^University of Dundee, Dundee DD2 4BF, UK; ^9^Quintiles GmbH, 63263 Neu-isenberg, Germany

## Abstract

The Learning Health System (LHS) describes linking routine healthcare systems directly with both research translation and knowledge translation as an extension of the evidence-based medicine paradigm, taking advantage of the ubiquitous use of electronic health record (EHR) systems. TRANSFoRm is an EU FP7 project that seeks to develop an infrastructure for the LHS in European primary care.* Methods*. The project is based on three clinical use cases, a genotype-phenotype study in diabetes, a randomised controlled trial with gastroesophageal reflux disease, and a diagnostic decision support system for chest pain, abdominal pain, and shortness of breath.* Results*. Four models were developed (clinical research, clinical data, provenance, and diagnosis) that form the basis of the projects approach to interoperability. These models are maintained as ontologies with binding of terms to define precise data elements. CDISC ODM and SDM standards are extended using an archetype approach to enable a two-level model of individual data elements, representing both research content and clinical content. Separate configurations of the TRANSFoRm tools serve each use case.* Conclusions. *The project has been successful in using ontologies and archetypes to develop a highly flexible solution to the problem of heterogeneity of data sources presented by the LHS.

## 1. Introduction

The Learning Health System (LHS) describes an approach to improve healthcare that is solidly founded on the creation and use of knowledge; “health” as opposed to “healthcare” is sometimes used to emphasise the role of consumers as cocreators and users of health knowledge [1]. The development of the LHS is a natural outcome of the evolution of evidence-based medicine (EBM). Based on the greater utilisation of electronic health records (EHRs) and on novel computing paradigms for data analysis, the LHS provides potential solutions for the glacial slowness of both the traditional research process and the research translation into improved care [2].

EBM is focused on generating medical evidence and using it to make clinical decisions. The highest level of evidence, level 1 evidence of the effectiveness of a healthcare intervention in EBM, consists of a meta-analysis of randomised controlled trials (RCTs) [3]. However, RCTs are complex and extremely expensive, the result being that much of healthcare remains unsupported by high quality evidence. Furthermore, RCTs themselves are prone to bias and manipulation in the choice of eligible subjects, comparators, and outcome measures [4]. One solution has been to carry out light touch and simple, termed “pragmatic” RCTs with very inclusive eligibility criteria and followup via routine data collection. It is those kinds of RCTs that lend themselves most to incorporation into a LHS.

There is also potential to replace RCTs with analysis of routine data, using techniques such as instrumental variables and propensity scores to control for bias [5]. Much future research is needed to define when routine data could be a sufficient answer to a problem and when an RCT is required. Furthermore, healthcare practice is not solely limited to interventions, but diagnosis and prognostication play essential parts and are underpinned by prospective cohort evidence. Again, routine data could play a significant role in replacing time-consuming and costly cohort designs.

Primary healthcare is the first point of contact with health services of patients with undifferentiated problems and also provides continuing care for patients with chronic diseases and follows families from “cradle to grave.” These functions present a particular problem for EBM. The vast majority of research, be it diagnostic or intervention based, takes place in specialist centres and in highly selected populations [6]. Diagnostic features are not portable across populations with different prevalence and spectrum of disease. Likewise, patients in RCTs are younger and fitter, take fewer drugs concurrently, and have less comorbidity than typical primary care populations. Therefore, many RCTs suffer from limited external validity [7].

Even if appropriate research evidence exists, it is unlikely to be available at the point of care. Early formulations of EBM typically applied to the highly motivated clinician who formulates questions during clinical practice and searches for evidence. Indeed, Professor Sackett's team at Oxford developed an “evidence cart” for ward rounds, with a copy for MEDLINE and a projector to assist in this process in real time [8]. Over the subsequent years, the process of knowledge translation has become formalised: guidelines are explicitly built on systematic reviews of the best available evidence and are refined down to a series of statements to support clinical care, with an associated level of supporting evidence and strength of recommendation [9]. However, even in countries like the UK, where a national agency (National Institute for Health and Care Excellence) is funded to carry out this process, guidelines may only be updated once in a decade. Increasingly, the number of potential guidelines applicable to a given patient at a given point on the care pathway becomes a problem of memory and prioritisation for the clinician, let alone the patient. The LHS offers a potential means of using highly advanced electronic triggers to help with advising when one treatment or diagnosis is favoured. It should also be possible to reintroduce patient choice by explicit weighting of options using patient-derived outcome data.

The LHS concept is still in its infancy, and much needs to be done to explore and demonstrate the potential for using an advanced digital infrastructure to support the LHS. The FP7 TRANSFoRm project (http://www.transformproject.eu/) was funded via the Patient Safety Stream of ICT for Health. Efficient research design and knowledge translation are a core underpinning of safe clinical practice. It is not good enough to simply avoid error, defined as care that falls well below the average standard, but clinicians should be seeking optimal care for their patients. The LHS, at its barest essential, is all about promoting optimal care. The TRANSFoRm project aimed to develop and demonstrate methods, models, standards, and a digital infrastructure for three specific components of the LHS:

1. genotype-phenotype epidemiological studies using multiple existing primary care and “biobank” genomic datasets;
2. RCTs with both data and trial processes embedded within the functionality of EHRs and the ability to collect Patient Reported Outcome Measures (PROMs) on demand;
3. decision support for diagnosis, based on clinical prediction rules (best diagnostic evidence) and fully integrated with a demonstrator EHR system.

## 2. Methods

Each specific clinical “use case” (shown below) served four purposes: initial requirements elicitation; detailed modelling of infrastructure and required data elements; design of concurrent validation and evaluation studies; and final clinical demonstrations. 21 partner organisations in ten EU member states took part in the project, over five years. At the time of writing, the project has 11 months to run and the final evaluation and clinical studies are about to commence.

*TRANSFoRm Use Cases*

*Diabetes Use Case*. The aim of the Diabetes use case is to enable a distributed query to look for eligible patients and extract data from multiple federated databases. In the pilot study, the query will define patients and data to support analysis of the relationship between well-selected single nucleotide polymorphisms (SNPs) in type 2 diabetic patients and the response to sulfonylurea.

*GORD Use Case*. The aim of the GORD use case is to investigate the effectiveness of on demand versus continuous use of proton pump inhibitors on reflux symptoms, quality of life, and self-rated health in patients with gastrooesophageal reflux disease in primary care. The study will be conducted in five localities (UK: two vendors, Poland, Netherlands, and Crete) and it will aim to recruit, randomise, and follow 700 patients at 40 primary care centres using the clinical trial application.

*Diagnosis Use Case*. The aim of the diagnosis use case is to provide integrated point-of-care decision support for patients presenting with chest pain, abdominal pain, and shortness of breath.

TRANSFoRm aims to produce a highly flexible infrastructure that presents the lowest possible barriers to entry for EHR systems and datasets, but at the same time it makes the maximum use of the existing data standards and methods for managing heterogeneity, both structural and terminological, between data sources. A basic principle of the TRANSFoRm project was to use available standards and models as much as possible and integrate them into the TRANSFoRm infrastructure. It was decided early on in the project that TRANSFoRm would take a model-based approach, using 4 models to capture (1) clinical meaning, (2) research meaning, (3) provenance, and (4) diagnostic meaning. The latter is essentially a subset of the clinical model, but it was modelled separately for efficiency. The archetype approach of constraining one model against the other, in a two-level design (clinical and research), was used to describe data elements [10]. Where available, existing tools for building and maintaining models as an ontology were used, although we presented a novel use of LexEVS, which we employed to support both structural and semantic models [11].

Clinical concepts were modelled using an ontology (termed the Clinical Data Information Model, CDIM) [12]. Additional semantic detail for data elements was expressed by using LexEVS to support binding of terminology terms to CDIM expressions. For representation of research processes, we extended an existing domain model, the Primary Care Research Object Model, adding objects primarily in the clinical area [13]. The resulting Clinical Research Information Model (CRIM), in conjunction with CDIM, enabled a two-level archetype to be defined for each required data element in the use cases. In order to define case report forms and study designs for the RCT, we used the CDISC ODM and SDM standards, but adding an archetype approach for the description of the data element “payload” [14].

The intention from the outset with TRANSFoRm was that all models would be published, standards would be reused and adapted as required, the software would reuse the existing open source components, if available, and all TRANSFoRm software components would be made available as open source tools under an “Apache” license. We believe that the value lies in the data and the knowledge generated from it and that amortizing the infrastructure can only act as a potential barrier for realising the value of the data/knowledge.

Evaluation of TRANSFoRm will consist of a technical validation of the TRANSFoRm tools and three clinical and sociotechnical evaluation studies. For the DSS, an evaluation of the system, integrated with the In Practice Systems Vision 3 EHR system, is underway. General practitioners are conducting a simulated clinical session with actors simulating patients presenting with carefully prepared test problems. This is a within-subjects design, with the cases solved first without and then with the DSS and the primary outcome being accuracy. We also measure usability and amount of information coded into the EHR. The Diabetes use case is being evaluated on the basis of performance, as judged by users, of the system in selecting and extracting data from five databases. Accuracy of selecting eligible patients by users employing the TRANSFoRm Query Workbench will be measured. The GORD (gastrooesophageal reflux disease, a disorder caused by the retrograde flow of gastric contents from the stomach into the oesophagus, causing symptoms and/or mucosal damage) study is being conducted as a full clinical RCT (individual subjects randomised) with a nested evaluation study. Principal outcomes of the clinical study are symptom profiles and quality of life measured by PROMs (Patient Reported Outcome Measures) collected on smartphones via a dedicated TRANSFoRm mobile data collection app. The sociotechnical evaluation is a nested cluster trial and will compare recruitment rates, completeness of data, and costs of the TRANSFoRm system compared to usual practice, in this case, a simple web form for the clinical measures and paper questionnaires for the PROMs. The results of the three TRANSFoRm evaluation studies will be available in late 2015.

## 3. Results

The TRANSFoRm software ecosystem is comprised of a set of generic middleware components that provide essential shared functions for the LHS applications built in TRANSFoRm, namely, secure data transport, authentication, semantic mediation, and data provenance (with respect to processing of data within TRANSFoRm). As LHS is characterized by routine production, transformation, and dissemination of data and knowledge, secure channels and reliable authentication are necessary to ensure confidence and buy-in by the data owners. The data itself resides in a vast array of distributed repositories that vary both in structure and in terminology, making data interoperability a key requirement that TRANSFoRm delivers using a semantic mediation approach combined with the standard data connectivity module (data node connector: DNC). The DNC implements data interoperability, as well as managing workflow processes and data extraction for participating EHRs and data sources, as discussed in the next section. Different flavours of DNC operate in epidemiology and RCT use cases, as the RCT DNC has to support additional requirements of the RCT workflow. Data provenance capture in TRANSFoRm implements traceability, which is necessary both to support trust and transparency and to enable learning and improvement in LHS processes.

On top of these shared components, three application specific tools were built to support the use cases: epidemiological study query workbench, clinical trial monitoring tool, and a diagnostic support plugin for EHR systems.

**Figure 1:**
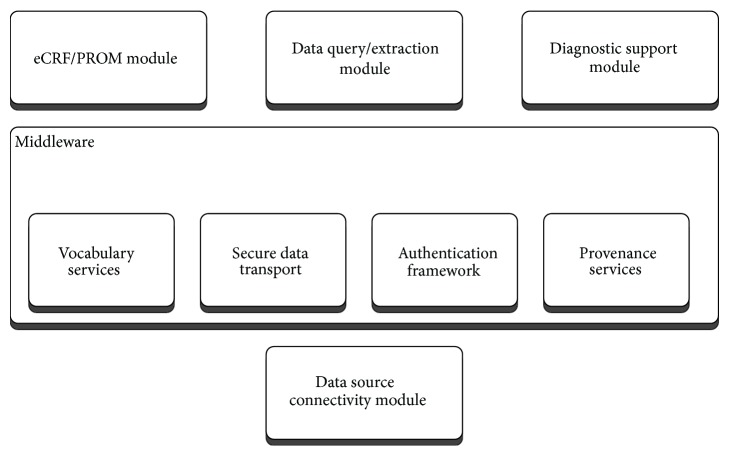
High-level software components.

The high-level overview of the software components is shown in Figure 1.

## 4. Epidemiological Study Application

**Figure 2:**
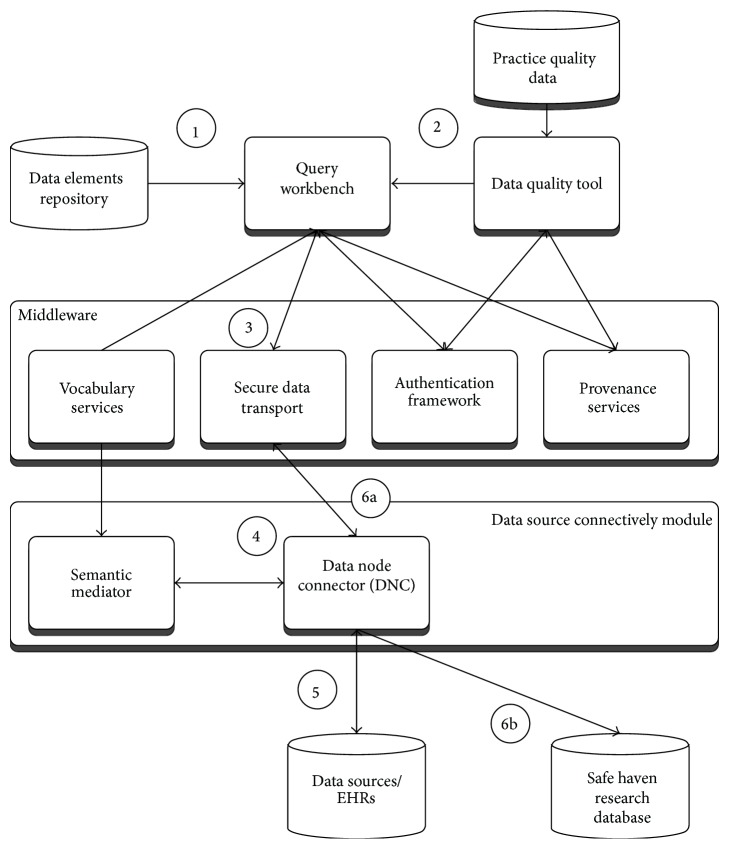
Epidemiological study configuration annotated with steps in the query process.

**Figure 3:**
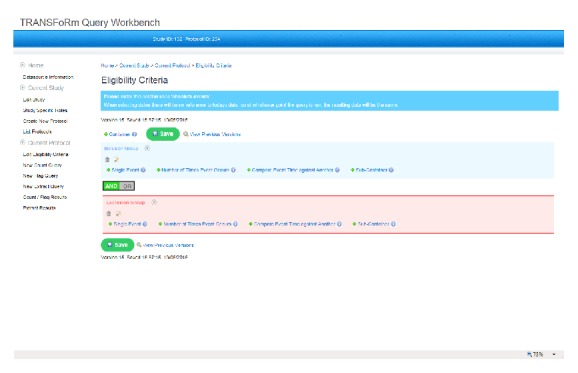
TRANSFoRm Query Workbench.

**Figure 4:**
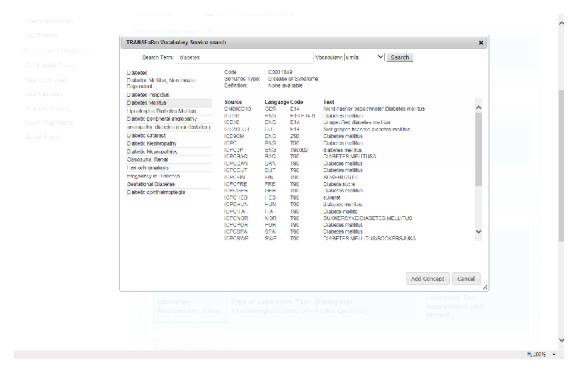
Concept search in TRANSFoRm Query Workbench.

The epidemiological study TRANSFoRm software configuration (Figure 2) is used in the genotypic-phenotypic T2D study use case and consists of tools for secure, provenance-enabled design and execution of eligibility queries and data extractions from heterogeneous data sources. Eligibility queries are formulated by the researcher in the query workbench (QWB) web tool (Figure 3) using model-based constructs (Figure 2, step 1). QWB users enter clinical terms into the system which then presents the user with a list of corresponding concepts from standard terminologies and classifications (Figure 4). The researchers are able to use a data quality tool, storing metadata about available practices and data that reside in them, to restrict the search to practices with a high registration percentage of the variables targeted in the study (step 2). The queries are dispatched to the data sources via the middleware (step 3) to the local data node connector. This is a TRANSFoRm component that sits at the data source and translates the generic CDIM-based query into a local representation using the semantic mediator component (step 4) and subsequently presents that locally interpretable query either to the data source directly or to a human agent for final approval (step 5), before returning the result. Three types of queries are supported: patient counts, flagging patients, and data extraction. Results of count and flag queries are sent back to the query workbench via the middleware (step 6a) and can be viewed by the researcher in the QWB web tool. The patient data extraction result is passed to a safe haven (step 6b), accessible only to the authorised researcher, using the appropriate secure data transport mechanism.

## 5. Clinical Trial Application

**Figure 5:**
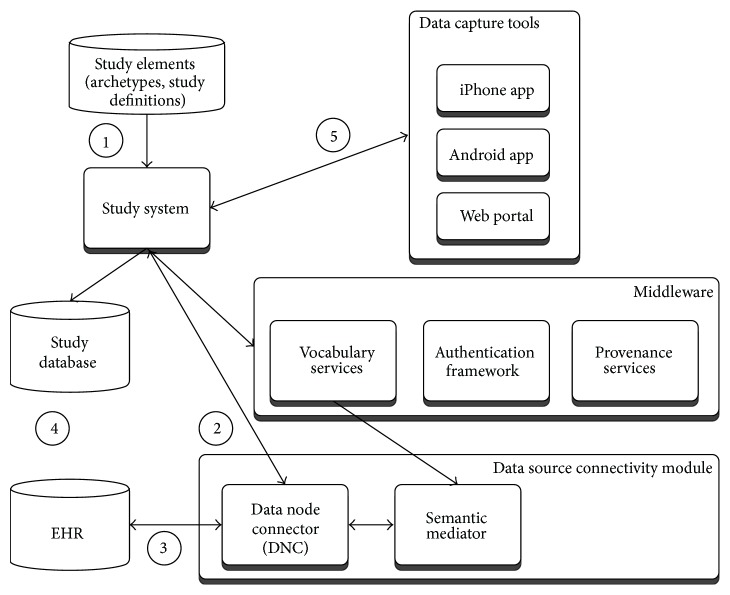
TRANSFoRm clinical trial configuration.

The clinical trial software configuration (Figure 5) is used in the GORD use case and consists of components needed for design, deployment, and collection of trial data, backed by provenance and secure authentication framework for researchers. The trial data collection is supported using electronic Case Report Forms (eCRFs) and Patient Reported Outcome Measures (PROMs). The former are filled in via a web browser by the clinician, while the latter are completed by the patients using either web or mobile devices. Also supported is the orchestration of data collection across multiple clinical sites where the trials are taking place.

The TRANSFoRm architecture delivers important components of clinical trials: patient eligibility checks and enrolment, prepopulation of eCRF data from EHRs, PROM data collection from patients, and storing of a copy of study data in the EHR. The key component of the architecture is the TRANSFoRm Study System (TSS) that coordinates study events and data collections, using HTML form templates with bound queries for preloading data from the EHR. The studies, represented using a custom extension of CDISC SDM/ODM standard, are loaded into the TRANSFoRm Study System (step 1). Whenever an interaction is required between the Study System and EHR, for example, eligibility checks or partial filling of eCRF forms form EHR data, a query is fired off to the EHR via the data node connector (step 2). As in the epidemiological study configuration, the DNC acts as a single point of contact of TRANSFoRm components and the local EHR. In addition to translating and sending queries to the EHR (step 3), the DNC acts as a web server that displays eCRF forms for the clinician to fill with study-required information not present in the EHR. Once completed, the form is submitted to both the study database and the EHR for storage considering requirements for eSource data use in clinical trials (step 4). The message protocol for this interaction is currently undergoing comparison evaluation with the IHE standards [17]. The PROM data is collected directly from the patients using web or mobile devices (step 5). The software configuration for the GORD study undergoes a formal Computer System Validation (CSV) process including qualifications for installation, operation, and performance to ensure that study system and study process have been Good Clinical Practice- (GCP-) validated prior to being employed in the GORD clinical trial use case. Because of the narrow connection between EHR and study system, part of GCP-validation is the assurance of data privacy and confidentiality of the personal patient data.

## 6. Diagnostic Support Application

**Figure 6:**
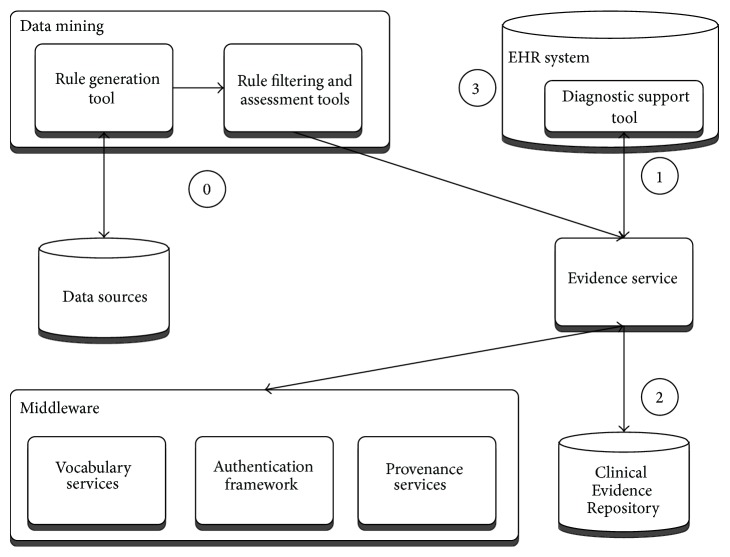
Diagnostic support configuration.

Diagnostic support software configuration (Figure 6: diagnostic support configuration) consists of tools for mining new rules from health data sources and managing their deployment into the knowledge base, upon which an evidence service is operating to drive a diagnostic support tool embedded into a local EHR system.

The primary function of the tool is to suggest to clinicians diagnoses to consider at the start of the clinical encounter based only on the existing information in the patient record and the current reason for encounter [18]. It also allows bottom-up input of observed patient cues (symptoms and signs), independent of associated diagnosis, or top-down drilling into and selection of cues supporting specific diagnoses.

**Figure 7:**
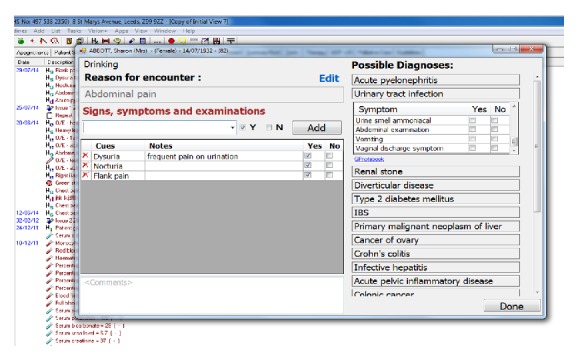
Diagnostic support tool implemented as a plugin to InPS Vision EHR system.

The rules used in the diagnostic process are generated by data mining tasks (step 0), which get manually curated and fed through the evidence service into the Clinical Evidence Repository. When the patient presents, the cues entered or selected are then used to dynamically rank the potential differential diagnoses (Figure 7). This is done by the DSS plugin embedded into the EHR, sending data to the evidence service (step 1), which queries the rules stored in the Clinical Evidence Repository (step 2), before sending the potential diagnoses back, annotated with levels of support and confidence for the presenting case. Upon exiting the tool, the coded evidence cues and current working diagnosis can be saved back to the patient EHR (step 3).

## 7. Conclusions

TRANSFoRm demonstrated how a Learning Health System can be implemented in European clinical research and practice. The full list of project outputs and the exploitation plan for each are shown in Table 1 and promoted via an open source model. TRANSFoRm will be a full participant in the European Institute for Innovation through Health Data and will make its tools and models available via the institute. In addition, we are internationally active as participants and promoters of the Learning Healthcare System. Via the LHS, we are publishing models, standards, and tools to the world research community. The UK serves as an exemplar of our business model, with multiple EHRs participating in the project as well as the Medicines and Healthcare Products Regulatory Agency, Clinical Practice Research Datalink (CPRD). CPRD currently extracts data from practices to a total population of 8 million and links them to 20 other health datasets. CPRD will be using the TRANSFoRm clinical trial tools, in conjunction with additional reworking by a commercial software vendor to create a full EHR-embedded clinical trial facility for the UK Clinical Research Network.

## Figures and Tables

**Table 1:** A table of outputs and exploitation plans.

TRANSFoRm output	Exploitation plan
(1) Privacy model: a “zone” model with an explicit method of graphically depicting the zones and operation of filters between zones	Published method [15]

(2) Provenance infrastructure: based on the Open Provenance Model [REF], each infrastructure component captures a provenance trace that enables reconstruction of an audit trail for any given data element	Published method [16]

(3) Clinical prediction rule ontology based web service	The diagnostic ontology has been made available as a public download in OWL format on the TRANSFoRm website (http://www.transformproject.eu/). A future project is required to extend the data beyond the three initial reasons for encounter

(4) Research data model	CDIM [12] and CRIM [13] have been published. A full description of the use of CDIM and CRIM in the construction of data node connectors will be published and made available on the TRANSFoRm website

(5) eCRF	Extension of CDISC ODM and SDM by the incorporation of archetypes with references to the CRIM and CDIM models will be published and discussions are ongoing with CDISC regarding future incorporation into the standards. A reference implementation of the clinical trial system will be maintained within the European Institute. At present, individual archetypes have to be written by hand; discussions are in hand for the production of an archetype authoring tool

(6) Data federation	A reference implementation of the genotype-phenotype study system will be maintained within the European Institute. Search authoring tools will be available open source

(7) DSS integration	The DSS is currently integrated with the InPS Vision 3 system. Further work is required to move this to a data node connector/CDIM-based flexible system
